# The Influence of Culture on Occupational Therapy Practice in Jordan

**DOI:** 10.1155/2020/1092805

**Published:** 2020-06-29

**Authors:** Somaya H. Malkawi, Nisrin S. Alqatarneh, Elaine K. Fehringer

**Affiliations:** ^1^Occupational Therapy Department, Rehabilitation Sciences Faculty, University of Jordan, Queen Rania Al Abdullah St. Amman, 11942, Jordan; ^2^Occupational Therapy Department, Faculty of Applied Health Sciences, Hashemite University, Zarqa 13133, Jordan; ^3^Department of Physical Therapy, Samford University, 800 Lakeshore Drive, Birmingham, AL 35229, USA

## Abstract

**Background:**

Occupational therapy's origins draw from Western culture, values, and beliefs which may impact the application of traditional occupational therapy practice in non-Western cultures.

**Purpose:**

This study explored how occupational therapists in Jordan facilitate occupational therapy practice within Islamic Eastern culture.

**Method:**

A phenomenological approach was used in this study. Semistructured interviews were conducted with eleven occupational therapists that work in Jordan and have at least two years of experience. Data were analyzed using thematic analysis methods.

**Results:**

Three central themes emerged: impact of Jordanian culture and Islamic beliefs about independence and disability on occupational therapy practice, the therapists' notions of ideal occupational therapy practice vs. daily reality, and challenges posed by workspace and the availability of equipment.

**Conclusion:**

This study highlights the growing need to translate and expand the core values of occupational therapy to align with cultures in non-Western countries and cultures.

## 1. Introduction

Occupational therapy (OT) identifies engagement in occupation as essential to human beings' existence [1]. A highlighted emphasis of OT in the literature is its focus on health, well-being, and participation in life through engagement in occupation [2]. Occupational therapists (OTs) are educated to assist clients in learning or relearning how to engage in meaningful occupations. While acknowledging this aim, it is also imperative to recognize and account for the sociocultural contexts in which the occupations take place [3]. Due to criticism of the individualistic orientation of the OT profession towards clients' engagement in occupation [4, 5], the World Federation of Occupational Therapists (WFOT) [6] urges the profession to actively consider the effect of culture on the delivery and reception of OT services.

Culture is defined in many ways. Mumford (p. 157) defined it as a complex whole which includes knowledge, beliefs, art, morals, law, customs, and any other capabilities and habits acquired by man as a member of society [7]. Lim more simply described culture as being comprised of traditional beliefs and social practices that lead to rules for social interaction within a particular locality or social group [8]; Iwama defined culture through an OT perspective as “shared spheres of experience and the ascription of meaning to objects and phenomenon in the world” [9] (p. 184).

Occupational therapy's philosophical foundation was initiated in the Western world using Western cultural assumptions to formulate the core concepts of the profession [10], thus creating a dominant cultural perspective within the models and theories favoring Western perspective [11, 12]. When using these core concepts, models, and theories within non-Western communities, occupational therapists are faced with challenges to bridge a gap in the fundamental understanding of their profession [11, 13, 14].

Most studies of healthcare practice focus on interventions typically used with clients from Western cultures. In contrast, little attention has focused on the influence of non-Western cultures and their impact on professional practice, particularly Arab cultures [15]. The perspectives of non-Western OTs and their clients are not sufficiently studied to present the impact of using Western-based models and OT concepts [16]. Al Busaidy and Borthwick discussed the impact of Arabic and Islamic culture on OT practice in a study that explored the importance of cultural beliefs and values to the practice of OT in Omani society [15]. They found that Omani clients' beliefs about religion, family, and gender roles have an impact on practice and pose challenges to therapists' ability to apply learned knowledge that originated from Western cultures to the practice of OT in their Omani culture.

For historical reasons, occupational therapy concepts and knowledge are conceptualized in the English language, requiring translation into other languages; however, aside from the cost and efforts to translate core concepts, the assumptions behind some of the profession's main concepts, theories, and models are often alien and incompatible with non-Western cultures [17–19]. This is an issue that is relevant to all non-English speaking cultures, as discussed by Brea et al. in relation to European languages [20].

On the other hand, teaching in OT programs based on an English language curricula is one of the main challenges non-Western academics face in transmitting fundamental OT concepts. The concepts are first learned in English, then translated into their native languages in practice [18]. This is the current situation in all three OT programs in Jordan where OT education is mainly taught in the English language [21]. In fact, the impact of the language begins with the profession's name itself (occupational therapy), as the word “occupation” in Arabic is used mainly to indicate “job or employment,” thus causing constant confusion with clients who are not familiar with this new profession in Jordan [22].

Occupational therapy as a profession was established in Jordan in 1989 and is currently being taught within three different Jordanian universities. About 42% of Jordanian OTs are employed in public or military hospitals, while 37% work within pediatric or school settings [21]. The most recent study regarding awareness of the OT profession in Jordan concluded that the OT role is still not well recognized among clients or allied health professionals. This was attributed to several factors such as the ambiguity and confusion related to OT concepts and definitions [22].

Criticism in the literature has also been raised towards some of the prevalent OT models as they do not consider non-Western culture. Attempts to employ typical Western intervention can lead to stereotypical assumptions about clients such as dependency, malingering, or noncompliance. When utilizing some of the assessments written to coordinate with these models, there is no guidance for how to consider adaptation for use with clients of diverse cultures [14, 21].

Most commercially available assessment and evaluation tools are based on Western cultural norms and founded on core concepts that might be inappropriate within other cultures [16]. For example, Western cultures focus on individualism, autonomy, competence, and mastery which does not translate into many non-Western cultures where interdependence and strong family involvement are deeply held cultural and religious values [16, 17]. Similarly, many cultures do not attribute the same meaning or value to Western notions of occupation that center around the act of “doing” and exerting control over the environment [11, 14]. Moreover, in Western cultures, there is a value for balance between work and leisure activities, whereas in some non-Western cultures, work and productivity are highly prized and leisure activities are generally ignored [10, 13].

Although there is no literature linking religion as a motivator or hinderer to occupational therapy, an Omani study which looked into an Arab-Islamic culture similar to Jordan found that clients considered their disability as being from the will of Allah [15]. Clients accept the disability, and thus, recovery from a disability is often viewed as only a matter God can determine. This cultural and religious perspective could easily be interpreted by Western culture as client passivity. The family structure, which is also related to the Islamic religious values within the Omani culture, was found to be more cohesive than in Western cultures. This observation was supported in a study in Singapore which also looked into another Muslim culture [15, 23]. In fact, it is common within collectivist cultures to value family duties and responsibilities to nuclear and extended family more than in some Western cultures; in these cultures, adults often live very separate lives from their family of origin and rely on healthcare professionals to provide services to the sick or disabled [13]. Occupations are culturally influenced; therefore, it is imperative that cultural practices, rituals, values, and beliefs be considered when therapists choose activities and media to present during treatment to clients [24]. Activities of daily living, which are a primary focus of traditional occupational therapy, are inherently influenced by cultural norms, but little literature exists describing the linkage between culture and client acceptance of occupational therapy intervention [25].

Occupational therapists, whether they are practicing in Western or non-Western environments, need to consider culture as a part of the therapeutic process. It is vitally important that occupational therapists account for culture so that truly meaningful intervention is designed and offered as part of the therapeutic process. While representing the perspective of non-Western occupational therapists is valued in the literature [4, 16, 19] to enact change within the profession towards a more culturally relevant occupational therapy, the voices of Arab-Muslim therapists must be heard. At present, there are very few studies exploring the perspectives of Arab occupational therapists towards their work within an Arabic-Muslim culture. The purpose of this paper is to explore and describe the lived experience of Jordanian OTs and to describe the influence of an Arabic-Islamic culture on occupational therapy practice in Jordan.

## 2. Materials and Method

This qualitative study utilized a phenomenological approach to uncover, explore, and describe the complex experiences of occupational therapists living and practicing in Jordan [26]. The researchers' position as Jordanian occupational therapists themselves provided them with an insider view of the lived experiences of the participants. This became valuable as researchers translated the transcripts from Arabic to English, using their background to interpret participants' words while maintaining the meanings which could be otherwise lost in translation.

### 2.1. Participants

Participants were contacted through an advertisement on social media and email. Purposeful sampling was used to select participants who had received their OT training in Jordan, were working as an OT for a minimum of two years at a Jordanian hospital or center, were available to participate in an in-depth audiotaped face-to-face interview, and were able to communicate an expressed opinion in an articulate and reflective manner [27]. The data were derived from a series of semistructured interviews of 11 occupational therapists working in several hospitals and centers around Jordan.  Table 1 shows other demographic description of the participants.

### 2.2. Data Collection

The interview protocol was constructed based on thorough readings of the literature. Open-ended questions were developed, and a pilot interview was implemented before finalizing the interview protocol. The principal investigator interviewed occupational therapists either at the hospital/center or university research office. Participants signed a written informed consent and completed a basic demographic information form prior to starting any interview. All interviews were conducted in Arabic to eliminate confusion related to speaking a second language. Audio recordings were transcribed verbatim within a week of the interview and then translated into English by the researchers. The translation process was carefully discussed to ensure participants' views were reflected accurately.

Participants were asked to describe their perception of the influence of Jordanian culture on OT practice. Therapists were queried about their experiences in providing intervention; probing questions were utilized to uncover their experiences by asking them to give more examples. The questions delved into challenges faced when proposing intervention. The participants were asked to describe whether they viewed Jordanian culture as support or hindrance to the practice of occupational therapy (see Appendix A for the interview questions). The interviews lasted 25 to 35 minutes, and data were collected between January and June 2017.

To ensure credibility and trustworthiness, the research team comprised of the primary investigator and two occupational therapists met several times during the data analysis process. Moreover, several strategies were implemented to increase the rigor of the data collection and analysis such as reflective journaling and field notes were kept to document researchers' thoughts and reflections during each step of the research, member checking with two participants completed after the transcription of the interviews and after the construction of the themes. This process provided a level of credibility to the findings [28].

This study was approved by the Institutional Review Board at the Deanship of Academic Research at the University of Jordan.

### 2.3. Data Analysis

The analysis of the interview data was guided by a thematic analysis which is commonly used in phenomenological studies to manage the thick descriptions attained from data. Significant statements were analyzed, meaning units generated, and the essence of meaning identified yielded comprehensive and clear interpretations.

Data analysis began with the first interview until saturation was reached. The final format of coding was analyzed by the collection of repeated information. Common encounters became clear; these experiences were grouped into categories which were collapsed into major experiences shared by the participants. Ultimately, three overarching themes emerged. Throughout data collection and analysis, a reflective journal was used by the two researcher assistants to document their thoughts and reflections during each step. Group analysis meetings were conducted between the researcher assistants and principal investigator to review emerging themes and reach consensus in thematic analysis. This process provided a level of credibility to the findings as systematic comparisons between emerging categories were made [28].

## 3. Results

The analysis revealed three central themes of cultural aspects affecting occupational therapy practice in Jordan: (1) the impact of Jordanian culture on OT practice, (2) ideal occupational therapy vs. reality, and (3) workplace challenges.

### 3.1. The Impact of Jordanian Culture on Occupational Therapy Practice

The first central theme described participants' perspectives on the impact of Jordanian culture on their ability to provide occupational therapy as they were educated. They mentioned the dominant reliance on the medical model in Jordan (which suggests that illness is recognized through systematic observation and testing and fixed through a set of accepted procedures), the impact of Islamic religion, the client's use of traditional treatments, and cultural emphasis on interdependence. The participants highlighted their clients' confusion towards the role of occupational therapy, and they perceived this uncertainty to be related to the clients' preference for medical treatments such as medications:

“Usually, clients in our culture prefer something related to medical model because of its quick results, they choose medications to decrease pain rather than doing an activity for an hour.” (S.A)

Often, when clients are offered alternative therapy options such as occupation-based interventions, the therapists reported client refusal to participate. The occupational therapists recounted that clients would request exercise or medication:

“Generally, clients do not accept occupational therapy and its nature. When I focus on Activities of Daily Livings (ADLs) or Instrumental Activities of Daily Living (IADLs), clients reject it and prefer exercises.” (T.S)

Some Jordanian clients were perceived to consider some occupation-based activities as “weird and shameful”:

“When you do leisure activities with clients who have psychotic illnesses, they feel like it is really weird and shameful.” (M.N)

The participants gave these descriptions to emphasize their frustration with the clients' reactions towards their work. The occupational therapists' responses in this section of the interviews reflected discouragement as they voiced that “clients do not accept occupational therapy and its nature.” There was a sense that the participants accepted rejection of their services as part of Jordanian culture.

When asked about using occupations such as ADLs in their sessions, one participant presented a commonly received reaction from their clients:

“They do not accept it at all; they said why [do] I have to learn how to dress in the clinic and front of strange people when I have someone to dress me.” (M.R)

This reaction was reported to be very common among clients regardless of which setting the participants practiced. They found their emphasis on independence in ADLs to be disregarded by their clients because the Jordanian cultural practice does not fit with the philosophy of occupational therapy:

“Occupational therapists in Jordan agree that our Jordanian culture limits our practice compared with the philosophy of occupational therapy that fits more in Western culture.” (S.A)

Furthermore, Jordanian clients are reported to still hang on traditional folk medicine beliefs when it comes to therapy. Habits and traditions in Jordan interfere with the occupational therapists' ability to apply what they learned in school.

“Some of the traditions that still used are olive oil and onions for healing instead of going to physicians to seek therapy.” (L.D)

Satisfying elder clients was also an obstacle. Accordingly, participants claimed that older clients do not accept occupational therapy interventions easily because they do not have leisure interests or employment which makes it difficult to develop treatment goals and design meaningful intervention.

“Older adults do not accept the use of some tools that look like games and they refuse to use it.” (T.A)

“Most of the cases I had treated were older adults who had CVA, they left their jobs and had no interests compared to adults who came to achieve specific goals.” (M.R)

Although the participants reported their struggles concerning the Jordanian culture and occupational therapy practice, they also discussed the positive impact of Islamic religion and family cohesion which distinguishes the Jordanian society. They reported not having to persuade the clients to implement the treatment because religion compels them to do so:

“Islam force people to take care of their bodies, so the clients collaborate with the treatment plan.” (T.A)

The participants also highlighted the availability of caregivers in the sessions to support intervention because of family cohesion.

“Most of the time I do not worry about finding a caregiver, they were always present.” (T.S)

The support of the family in the occupational therapy process was emphasized by the participants who worked with children, as they described the valuable sources of information and support parents provide the therapists.

### 3.2. Ideal Occupational Therapy versus Reality

The second theme is related to challenges the participants face when encountering the reality of occupational therapy practice in Jordan compared to what they learned in their education programs. The participants reported a clear gap between the “optimal way of occupational therapy practice” and the theoretical knowledge they have concerning occupational therapy practice:

“Yes, there is a big difference; we learned the optimal way of occupational therapy practice, but we find that we are very far from it.” (T.A)

“There is a big difference in tools, culture, and knowledge about occupational therapy. So the clients, institutions, and administrations sometimes do not accept our needs.” (S.A)

The participants spoke with particular emphasis about the challenge of using assessment and measurement instruments which are written in English, and the normative data are based on English-speaking clients, with their clients who spoke Arabic. They described attempting to translate every time in the simplest way for the clients but presented this as a major barrier:

“Often, it is hard to explain the assessment tools for the clients simply.” (S.A)

“When I find the appropriate assessment tool, I have to translate and explain it to the clients especially for older adults and unenlightened people.” (T.S)

At the same time, the participants described their coping strategies to bridge this gap, which focused on finding a compromise between their clients' preference for the medical model, and the occupational therapists' efforts to use occupation-based, client-centered practice as much as they can:

“In fact, I always try to get clients' satisfaction. I start with exercises then gradually integrate functional activities including ADL for patients who do not accept functional activity.” (M.R)

The participants perceived their efforts to develop their knowledge and skills as one way of coping with the struggles they face:

“I improved myself by studying, practice, training, and searching for more information as much as I can.” (L.N)

### 3.3. Workplace Challenges

This theme included difficulties related to the daily challenges participants encountered in their workplace. They discussed the limited resources they had available for OT work including culturally relevant assessment and measurement instruments and intervention items, such as splinting materials, heat guns, and ADL equipment such as sock donning aids. One therapist worried that she would not be able to fully assess and document clients' needs and progress or provide therapy services in the manner in which she had been educated.

“There is a lack of essential therapy equipment and tools in the hospital due to limited funding/budget. No matter how skilled I maybe, I will never be able to work at full capacity if there is a lack of tools.” (T.S)

This challenge caused feelings of frustration by the participants who emphasized the importance of such tools. They perceived the lack of assessment tools and intervention equipment as impeding their work. It is worth noting that these reported challenges were mentioned by participants regardless of their practice settings (i.e., private clinic or publicly funded). The lack of therapy supplies and equipment in occupational therapy clinics might potentially lead to therapeutic inefficiencies, diminished results, and possible delay of improvement among patients. Notably, as the participants talked, it became apparent that the lack of basic supplies and space impacted the morale of the therapists.

The participants also mentioned their struggle to collaborate with other members of the medical team; this was discussed by most of the participants as one of the issues impacting their work. The participants reported the potential negative results of lack of cooperation between other members of the medical team and felt the lack of collaboration impacted the quality of the services:

“I'm unable to communicate and participate with the medical team members to develop an integrated treatment plan. Each of us works independently and sometimes [that] affects the quality of services we provide to our patients.” (T.S)

One of the reasons mentioned by the participants for this lack of collaboration was the misunderstanding of the role of occupational therapists by the other members of the team.

“[The] medical team does not know how occupational therapists work and what [is] their contribution to patients' health. They need to be educated more about our role in the rehabilitation team.” (T.A)

Physicians' misunderstanding of occupational therapy practice was evident in most interviews. What the occupational therapists wanted is basic communication with the physician and, most importantly, to be seen, understood, and taken seriously. The participants perceived that most other healthcare providers did not understand the occupational therapy role which had a negative effect on their ability to execute the intervention plan.

Some parents told me: “I spent an hour here for those useless games that you do with my child.” They do not know that our treatment with pediatrics is based on playing. (H.M)

At the same time, participants described limitations related to the facilities available for their use, which impacted the type of intervention they choose, especially when privacy was required:

“Some activities exist that require a high level of privacy, especially when it is related to gender issues or toileting.” (T.S)

While the participants were aware of the importance of providing their clients with privacy when they worked on activities such as toileting, they described situations where such privacy was impossible:

“It is always difficult to give the patient a suitable activity because no private place exists for one-to-one therapy. In fact, in the same therapy room, three different clients with different goals and occupational therapists are usually present.” (T.A)

## 4. Discussion

The overall results indicated that occupational therapy practice in Jordan is influenced by the population's values and culture and the activities that clients prefer and are willing to perform. The discussion that follows highlights the unique practice of occupational therapy in Jordan and is considered in the context of the three themes presented in the findings.

### 4.1. Promoting Western Occupational Therapy Ideology within Jordanian Culture

The participants explained their struggles to introduce their profession to their clients. This barrier appears to be linked to the dominant medical model, as clients state they “prefer exercises” to occupation-based interventions, which were perceived as meaningless and with less therapeutic potential. The rejection of such interventions, preferring medications and exercises, challenges the value of occupational therapy as a profession in Jordan. Participants talked about other lack of knowledge of healthcare professions and the general public in Jordan about what the profession offers. We need to consider that the application of occupations as a therapeutic agent is associated with Western cultural origins. The fundamental, individualistic concepts of occupational therapy may not translate and resonate within the Arabic-Islamic culture [11, 12]. An example of this dissonance is the emphasis OTs place on independence in activities of daily living which are considered by Jordanian clients as “a waste of time” due to the value given to interdependence within this collectivist culture. If the client expects their family to complete or assist them in their daily activities, why would they value an intervention that focuses on their independence in these activities? Al Busaidy and Borthwick and Yang et al. described a finding with similar lack of interest in independence in ADLs and perception of the diminished value of occupational therapy interventions [15, 23]. Both studies were conducted within Muslim countries, and both described a common challenge when attempting to implement core occupational therapy concepts during interventions.

Iwama described a similar challenge within the Japanese culture, which resulted in the introduction of the Kawa model [13]. The framework is aimed at introducing a culturally diverse practice model for non-Western cultures. The gap between the emphasis on independence in activities of daily living within the occupational therapy profession and the cultural perspective towards interdependence is reported in the occupational therapy literature as one of the struggles occupational therapists face in their practice when working with clients from the non-Western cultural background [23, 24, 29].

Education programs would do well to discuss with future occupational therapists this disconnect between Arab-Islamic culture and traditional OT values and practice which arise from a Western perspective and culture. This provides an opportunity to engage students in conversations about developing theories and practice recommendations that align with their culture and client preferences.

The theme, “The impact of Jordanian culture on occupational therapy practice,” is unique to Jordanian culture and focuses on the applicability of occupational therapy in Jordanian culture. Participants identified some values and thoughts of their Jordanian clients that affect their practice, and these are not mentioned in any previous literature because it is linked to Jordanian clients. The medical model, for example, was preferred by clients because they perceived they would achieve quicker, more beneficial results than with occupation-based interventions. The clients' preference for directive treatment, which is prevalent in the medical model, might be the result of a cultural bias towards “expert knowledge.” This preference can also be seen in clients' being more accepting of treatments and settings where medical items and treatment equipment such as electrical stimulation units or weights are in plain view as opposed to interventions linked to occupation-based models which address occupational needs and utilizes the clients' environment. The clients' perceptions and preferences challenge occupational therapists' assumptions about what “real OT” is and require them to strike a compromise between their clients' desires and remaining faithful to the profession's core values. Some participants talked about beginning their sessions with preparatory methods more congruent with the medical model and then moving clients gently into purposeful or occupation-based interventions. The participants voiced this straddling of two models required more time and effort, both of which were in short supply in their heavy workload.

Additionally, participants voiced concerns and frustration that because occupational therapy is a relatively new profession in Jordan and is not well known or understood, most clients come to therapy without knowing what type of interventions to expect. When the therapists start to provide occupation-based treatments, clients are resistant or reject the services. The participants reported coping with this by taking the time to educate their clients about the nature and importance of occupational therapy before providing it.

The lack of knowledge about OT by clients and other healthcare professionals was reported in previous studies investigating job satisfaction of OTs in Jordan [21, 22]. Because there is a lack of knowledge about the scope of occupational therapy practice in Jordanian healthcare and culture, it is possible that this is causing some of the Jordanian OTs' work-related angst. Although these struggles are common in Western cultures too, in this study, they are shown to have a link to the lack of understanding about the role of OT in Jordan, which could be due to the gap between traditional, Western OT fundamental concepts and the cultural understanding of the value of OT within this Arabic-Islamic culture. According to the Omani study, some Western principles cannot be translated into Eastern culture [15]. Participants stated that there is a stigma in their culture about the use of occupations in therapy. The core of occupational therapy, as conceptualized by Western culture, is that we help others return to their previous level of function which is defined as allowing a person to meet the demands of the environment. These activities support a person's physical, mental, and social well-being. For some people, in some cultures, returning to their previous level of function may mean fully engaging in previous daily occupations. In Arabic-Islamic cultures, there is not necessarily that expectation, or even desire, to return to that prior level. If this is the case, then some clients refuse to be trained to return their ability to do basic activities of daily living such as dressing and bathing. Other clients expressed they consider it “weird or shameful” to engage with a nonfamily member in completing ADLs such as bathing and toileting.

Some of the confusion about the focus of OT and the role of the therapist is perpetuated by the term “occupation.” Translation of this term into Arabic alludes to work or vocation more than the intended meaning of engagement in meaningful activities. This confusion is evident in other languages as well and has been discussed in the literature as a function of language interpretation of foundational occupational therapy terms and core concepts [18, 20]. This is a commonly reported struggle in previous studies in Jordan related to awareness of the OT role [22]. It is also reflected clearly in this study by the participants' reported struggles to explain their work to their clients and their colleagues.

At the same time, the cultural perspective towards a “sick person” in Jordan leans towards providing care for the sick and supporting them rather than encouraging their independence. This perspective is common within collectivist cultures as discussed by previous research, where interdependence is valued more than independence and autonomy [5, 13, 14]. Thus, clients frequently view the efforts of an occupational therapist to help them become independent in ADLs as “useless” and a waste of time when someone else in the client's family would be doing these activities for them.

Some Jordanian clients still cling to traditional beliefs about using homemade items for healing, and participants talked about having to persuade their clients about the benefits of their treatments. This finding mirrors the views of Omani clients [15]. Jordanian OTs stated that it is especially hard to satisfy the elder clients that come to therapy who want and expect to have their affected part returned to normal by the use of equipment and exercise; they frequently refuse any type of occupation-based interventions and consider these interventions useless because they have no jobs or leisure interests.

### 4.2. Finding a Compromise between Ideal Occupational Therapy and Reality

Although there were a lot of barriers facing the therapists in their practice, they did successfully cope with some of those challenges. This point considers the second theme of this study. According to the previous literature review, occupational therapy theories and concepts are based on Western culture which undeniably affects occupational therapists' practice in Jordan. These participants experienced the gap between the optimal way of practice that the therapists learned about in the universities and the real situation of practice here in Jordan. Occupational therapy is a relatively new major in Arabic universities and, therefore, a fairly new profession in Arabic Islamic countries [22]. Because of this, most healthcare facilities lack many of the tools and equipment of our trade. If assessment tools to quantify patient performance are available, most of them are only available in English, which requires the therapist to translate and explain the procedures to the clients in the simplest way. Although in the past years Arab, and in particular Jordanian, researchers worked on translating assessment tools into Arabic [30–32], their work is still limited to using available Western-based assessment tools rather than producing tools that address culturally specific occupations such as prayer or collectivist occupations.

The participants explained coping with these challenges by merging their priorities in the therapeutic plan with the client's priorities to engage clients and achieve good outcomes. They displayed creativity in using available tools to gather assessment data and deliver relevant interventions. Similar strategies were used by Omani OTs who looked for a compromise between their profession's Westernized perspective and their own Arabic culture [15].

These participants identified challenges and obstacles in the relationship between occupational therapy and Jordanian culture but articulated some positive and supportive sides in the Jordanian culture. They viewed strong family cohesion as positive. This supported the findings of the Omani study in which most families felt it was their responsibility to take care of the disabled family member [15].

Another identified positive is the Islamic religion which compels the client to take care of their health and body which sometimes leads to a commitment to the therapy process. On the other hand, according to Omani study [15], in the Arab world, one religious belief is a form of fatalism; illness, injury, disability, or other life events are viewed as the “will of Allah,” and within Islam, patients consider recovery as exclusively a matter for Allah to determine. This religious belief may encourage passivity in the rehabilitation process. This religious perspective is also revealed in other Islamic cultures [23] where OTs attempt to utilize the Islamic belief of a person's responsibility to seek medication and treatment to encourage their clients to participate in OT sessions. On the other hand, the participants in this study emphasized their clients' expectations of family members to assist them in ADLs; this can be explained by the Islamic principle of supporting the weak and sick as part of the community responsibility, as derived from the teachings and directions from Prophet Mohammad who said: “The similitude of believers in regard to mutual love, affection, feeling, is that of one body; when any limb aches, the whole body aches, because of sleeplessness and fever” (32, 6258) [33].

In this study, the participants explained the different perspectives they encounter which are related to cultural or religious understandings and the participants' responses revealed their efforts to find compromises and utilize their own cultural understandings to support their clients. These efforts, while linked here to the Jordanian culture, do have resonance with at least two Muslim cultures as revealed in one study from Oman [15] and another in Singapore [23]. More research from other Muslim and Arabic cultures is needed to establish more widespread evidence for these efforts.

### 4.3. Occupational Therapy Interventions Hindered by Limited Resources

Participants reported paucity of occupational therapy specific assessment tools and therapeutic resources, as well as limited facilities allowing privacy for their clients, which the participants viewed to negatively impact their work. As they attempted to use occupation-based intervention, they were challenged within their work settings by the focus on medical treatment, namely, medications and exercise-based therapy. This is similar to the challenges non-Western occupational therapists reported in other countries [15, 23, 34] where occupational therapy is geared towards medically based or biomedical interventions to fit the dominant medical model and limite4.4d resources.

The Jordanian culture is based on Islamic teachings including those related to modesty and privacy. In Islam, the body of a person is covered to ensure decency, so OTs who are working on gender-related or toileting activities report a challenge to ensure their clients' privacy; they are in fact highlighting an issue that is directly related to Islamic-Arabic culture. This challenge is not discussed directly or sufficiently in the literature [23]. In this study, participants stated that the lack of privacy in facility spaces often eliminates the option of implementing interventions that address toileting, dressing, and other ADLs that require privacy for clients. Al Busaidy and Borthwick found a similar dilemma in their study of Omani culture particularly when dressing or toileting assessment and interventions that involved therapists and clients of the opposite sex [15]. A workaround that solved this cultural problem was to present a home program of care and advise family members of how to implement it.

### 4.4. Study Limitations

The participants' sample did not represent all geographic regions in Jordan with most of the participants from the north and middle of Jordan. A future study should include participants in south Jordan to capture viewpoints from all areas of Jordan. Moreover, not all practice fields were included. Only therapists working in neurophysical and pediatric rehabilitation were included; therefore, occupational therapists who work in mental health were not represented. We recommend including all practice fields in future research to achieve a more comprehensive view of occupational therapy practice in Jordan. Finally, this study does not consider clients' ideas or perceptions about occupational therapy and its applicability in Jordan. It is recommended that future research should include both therapists' and clients' perceptions.

### 4.5. Clinical and Research Implications

This study revealed a need to look further into the views of Jordanian occupational therapists and their clients regarding their understanding of the profession and its values. As the OT profession currently relies heavily on Western-based core values and theories, the application of those ideologies to non-Western countries needs to be studied further and the development of practice models relevant to Arab-Islamic culture is encouraged.

Because occupational therapy is still considered a new and often misunderstood profession in Jordan, there is an urgent need to provide policymakers and university programs with evidence to enact change in the profession's profile, in order for occupational therapy to develop and adapt to the cultural needs of the Jordanian community.

The study suggests a discrepancy between the focus of OT on ADLs and the actual expectations of Jordanian clients, who—according to the participants—are not interested in ADL-based interventions; this discrepancy might be related to the emphasis given to independence in ADLs which is derived from Western cultures, as opposed to the Jordanian culture where families are expected to help the sick members with their ADLs. However, further studies to explore this from the perspective of Jordanian clients are needed.

Lastly, the profession of OT needs to focus attention on the issue of cultural diversity as clients from different cultural backgrounds frequently require services within Western countries; thus, Western therapists are encountering clients from many different cultural backgrounds and they deserve to receive culturally relevant services.

## 5. Conclusion

Jordanian culture is influencing the practice of traditionally conceptualized occupational therapy by workplace settings, clients' values, and therapists' identity as respected clinicians. Although faced with these challenges, therapists do cope, draw on their creativity, and find ways to work within cultural obstacles to continue to advance the practice of occupational therapy in Jordan. They identified positive aspects of Jordanian culture such as religion and family cohesion that support the occupational therapy practice. This study highlights the growing need to translate and expand core values and models of practice of occupational therapy to align with cultures in non-Western countries and cultures.

## Figures and Tables

**Table 1:** Demographic characteristics of the participants (*N* = 11).

Variable	*n*	%
Gender		
Male	3	27.3
Female	8	72.7
Years of experience		
2-4 years	3	27.3
4-6 years	3	27.3
>6 years	5	45.4
Geographical location		
Amman (the capital)	7	63.6
Other cities	4	36.4
Area of specialization		
Pediatric	5	45.5
Others	6	54.5

## Data Availability

The qualitative data used to support the findings of this study are restricted by the University of Jordan ethics committee for the confidentiality of participants. Data are available for researchers who meet the criteria for access from the primary investigator.

## Appendix

### Interview Protocol

1. Have you found a clear difference between what you have learned about occupational therapy among the books and practice, what is it?
2. How did you deal with the difficulties that you face in the practice of occupational therapy? Have you adapted, and how?
3. What are the difficulties in assessing the patients?
4. What are the factors that affect the nature of the activity that you do with your clients?
5. What aspects of occupational therapy do you ignore? Why?
6. How could Arab culture support or hinder the practice of occupational therapy?
